# Chronic Zinc Deficiency Alters Chick Gut Microbiota Composition and Function

**DOI:** 10.3390/nu7125497

**Published:** 2015-11-27

**Authors:** Spenser Reed, Hadar Neuman, Sharon Moscovich, Raymond P. Glahn, Omry Koren, Elad Tako

**Affiliations:** 1 USDA-ARS Robert Holley Center for Agriculture and Health, Ithaca, NY 14853, USA; smr2@cornell.edu (S.R.); rpg3@cornell.edu (R.P.G.); 2 Division of Nutritional Sciences, Cornell University, Ithaca, NY 14853, USA; 3 College of Medicine, the University of Arizona, Tucson, AZ 85724, USA; 4 Faculty of Medicine, Bar-Ilan University, 8 Henrietta Szold St, Safed 1311502, Israel; hadarneuman@gmail.com (H.N.); sharonmosc@gmail.com (S.M.); Omry.Koren@biu.ac.il (O.K.)

**Keywords:** zinc deficiency, microbiota, dysbiosis, cecal microbiome, zinc biomarker

## Abstract

Zinc (Zn) deficiency is a prevalent micronutrient insufficiency. Although the gut is a vital organ for Zn utilization, and Zn deficiency is associated with impaired intestinal permeability and a global decrease in gastrointestinal health, alterations in the gut microbial ecology of the host under conditions of Zn deficiency have yet to be studied. Using the broiler chicken (*Gallus gallus*) model, the aim of this study was to characterize distinct cecal microbiota shifts induced by chronic dietary Zn depletion. We demonstrate that Zn deficiency induces significant taxonomic alterations and decreases overall species richness and diversity, establishing a microbial profile resembling that of various other pathological states. Through metagenomic analysis, we show that predicted Kyoto Encyclopedia of Genes and Genomes (KEGG) pathways responsible for macro- and micronutrient uptake are significantly depleted under Zn deficiency; along with concomitant decreases in beneficial short chain fatty acids, such depletions may further preclude optimal host Zn availability. We also identify several candidate microbes that may play a significant role in modulating the bioavailability and utilization of dietary Zn during prolonged deficiency. Our results are the first to characterize a unique and dysbiotic cecal microbiota during Zn deficiency, and provide evidence for such microbial perturbations as potential effectors of the Zn deficient phenotype.

## 1. Introduction

Zn, an essential nutrient for nearly all organisms, is most notably involved as a metal cofactor in hundreds of proteins within the human body [1,2]. In healthy adults, Zn is present in the amount of 2–3 g and is second only to iron (Fe) as the most abundant micronutrient [3,4]. Even mild deficiencies of this mineral can profoundly impact growth and development, as well as impede immune differentiation and maturation [5,6]. The spectrum of chronic Zn deficiencies has been recently estimated to affect around 17% of the population [7], with insufficient dietary Zn intake and/or poor bioavailability from food central to this condition [8,9]. Despite the high prevalence of Zn deficiency, accurate clinical biomarkers of Zn status are lacking [10,11]. To address this, a major initiative set forth by the World Health Organization, the International Zinc Nutrition Consultative Group, and others has been to promote the development of reliable Zn biomarkers. Although serum Zn is currently the most widely used biomarker of Zn status, inherent problems with its measurement and interpretation can significantly impact sensitivity and specificity for dietary Zn [11]. To that end, our group recently published evidence in this journal for a new biological indicator of Zn status, the linoleic acid: dihomo–γ–linolenic acid (LA:DGLA) ratio, which exploits the Zn–dependent rate–limiting step of erythrocyte fatty acid desaturation [12]. Yet, since no single reliable biomarker of Zn status currently exists, establishing a panel of biochemical indices, as is the case with functional Fe deficiency [13,14], may be necessary. 

Understanding the influence of the gastrointestinal microbiota on physiology may represent a novel area to also understand the effects of Zn deficiency on the host. Little is known about how dietary Zn contributes to the microbiota, and even less is known regarding the effects of chronic Zn deficiency on the gut microbial composition. Early work by Smith *et al.* [15] elucidated a role of the host microbiota in Zn homeostasis, whereby conventionally-raised (CR, Conventionally-raised) mice required nearly twice as much dietary Zn than did their germ-free (GF) counterparts. In the same study, an *in vitro* assay using radiolabeled ^65^Zn identified a *Streptococcus* sp. and *Staphylococcus epidermidis* able to concentrate Zn from the medium. In this study, GF animals also had a reduced cecal Zn concentration relative to their CR counterparts.

Recently, it was shown [16] that Zn competition exists in *C. jejuni* and other bacterial species in the host microbiota of CR versus GF broiler chickens (*Gallus gallus*). Under conditions of Zn deficiency, this might lead to the preferential growth of bacteria able to survive at low-Zn levels. Further, many recent studies have shown that prophylactic doses of Zn (as Zn oxide, ZnO) in various animal models increased the presence of Gram–negative facultative anaerobic bacterial groups, the colonic concentration of short chain fatty acids (SCFAs), as well as overall species richness and diversity [17,18,19]. Likewise, others have found a gut microbiota enriched in members of the phylum Firmicutes, specifically *Lactobacillus*, following ZnO administration [20]. Therapeutic levels of dietary Zn have been shown to alter the overall gut microbial composition of piglets leading to favorable changes in its metabolic activity [21,22]. Protective effects of Zn supplementation include modulating intestinal permeability (via proliferation of the absorptive mucosa) [23,24], reducing villous apoptosis [25], influencing the Th1 immune response [26], and reducing pathogenic infections and subsequent diarrheal episodes [23].

Although the gut environment is central to Zn homeostasis, and is affected by suboptimal Zn status, we know little about the effects of chronic dietary Zn deficiency on the composition and function of the gut microbiome. Therefore, the present study examined how a 4 weeks period of Zn deficiency affected the composition and genetic potential of the cecal microbiota in broiler chickens fed a moderately Zn deficient diet. A panel of Zn status biomarkers was measured weekly, and gene expression of a variety of Zn-dependent proteins was quantified from relevant tissues at study conclusion. Cecal contents were collected for SCFA quantification and for analyzing compositional and functional alterations in the microbiota. 

## 2. Experimental Section

### 2.1. Animals, Diets, and Experimental Design

Upon hatching, chicks were randomly allocated into two treatment groups on the basis of body weight and gender (aimed to ensure equal distribution between groups, *n* = 12): 1. Zn(+): 42 µg/g zinc; 2. Zn(−): 2.5 µg/g zinc. Experimental diets are shown in Supplemental Table 1. At study conclusion, birds were euthanized. The digestive tracts (colon and small intestine) and liver were quickly removed and stored as was previously described [12]. All animal protocols were approved by the Cornell University Institutional Animal Care and Use committee. 

### 2.2. Determination of Zn Status

Zn status parameters were determined as described in the Supplemental Materials and Methods.

### 2.3. Isolation of Total RNA

Total RNA was extracted from 30 mg of duodenal (proximal duodenum, *n* = 9) and liver tissues (*n* = 9) as described in the Supplemental Materials and Methods. Supplemental Table 2 shows the measured genes.

### 2.4. Cecal SCFA Analysis 

SCFA concentration was determined as described in the Supplemental Materials and Methods. 

### 2.5. 16S rRNA PCR (Polymerase Chain Reaction) Amplification and Sequencing

Microbial genomic DNA was extracted from cecal samples as described in the Supplemental Materials and Methods.

### 2.6. 16S rRNA Gene Sequence Analysis

16S rRNA analysis was performed as described in the Supplemental Materials and Methods.

### 2.7. Statistical Analysis

**Figure 1:**
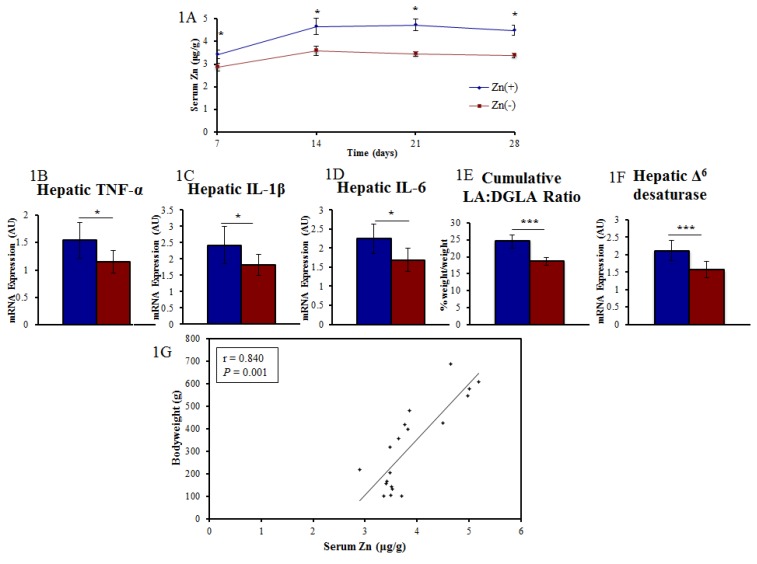
Measured Zn status parameters. (**A**) Day 7, 14, 21, and 28 serum Zn levels were significantly different between treatment groups (*n* = 12, * *p* < 0.05, ANOVA); (**B**–**D**) mRNA gene expression of hepatic tissue excised at the conclusion of study (*n* = 12, day 28, * *p* < 0.05, ANOVA); (**E,F**) Additional Zn biomarkers utilizing erythrocyte fatty acid composition (*** *p* < 0.001, *n* = 12); (**G**) Linear correlation between bodyweight and serum Zn on day 28.

Biomarkers of Zn deficiency (Figure 1) are presented as means ± SEM. ANOVA was performed to identify significant differences between the means of the experimental groups of birds, unless otherwise stated. Spearman’s correlation was used to assess significant associations between bacterial groups and biomarkers of Zn status. False discovery rate adjusted *p*-values were calculated for comparisons of taxa. *p* < 0.05 was considered significant. All statistical tests were two–tailed and were carried out using SAS version 9.3 (SAS Institute, Cary, NC, USA).

## 3. Results 

### 3.1. A Panel of Sensitive Biomarkers Defines a Marked Difference in Zn Status between Treatment Groups 

Results of the Zn status biomarkers used in this study were adapted from our recent publication [12]. Due to the lack of a singular marker of Zn intake and deficiency [27,28], we opted to use an array of biological indicators of Zn status-including growth (bodyweight), immunological (hepatic mRNA expression of cytokines), and physiological (tissue Zn, serum Zn, and the erythrocyte LA:DGLA ratio) parameters- to confirm Zn deficiency in the Zn(−) treatment group. As expected, these indicators (Figure 1A–F) were significantly different between animals receiving a Zn adequate semi purified diet ([29]; Zn(+), 42 µg/g Zn) versus those receiving a Zn deficient diet (Zn(−), 2.5 µg/g Zn). Relative hepatic mRNA gene expression of the pro-inflammatory cytokines IL-1β, IL-6, and Th2 dominant TNF-α were significantly reduced in the Zn deficient group, supporting a central role for dietary Zn in the production of cytokines and immunoregulation [30,31,32]. The chronic feeding of a Zn deprived diet resulted in a measureable Zn deficiency in the Zn(−) animals relative to their Zn(+) counterparts.

### 3.2. Gut Microbial Diversity of Zn Deficient Animals Resembles Physiologically Diseased Microbiomes

Cecal samples from the Zn(+) and Zn(−) treatment groups were harvested and used for bacterial DNA extraction and sequencing of the V4 hypervariable region in the 16S rRNA gene. The cecum represents the primary site of bacterial fermentation in *Gallus gallus*, with its resident microbiota highly diverse and abundant [33]. As in humans, Firmicutes are by far the dominant bacterial phylum in the *Gallus gallus* cecum, accounting for 70%–90% of all sequences [34,35].

**Figure 2:**
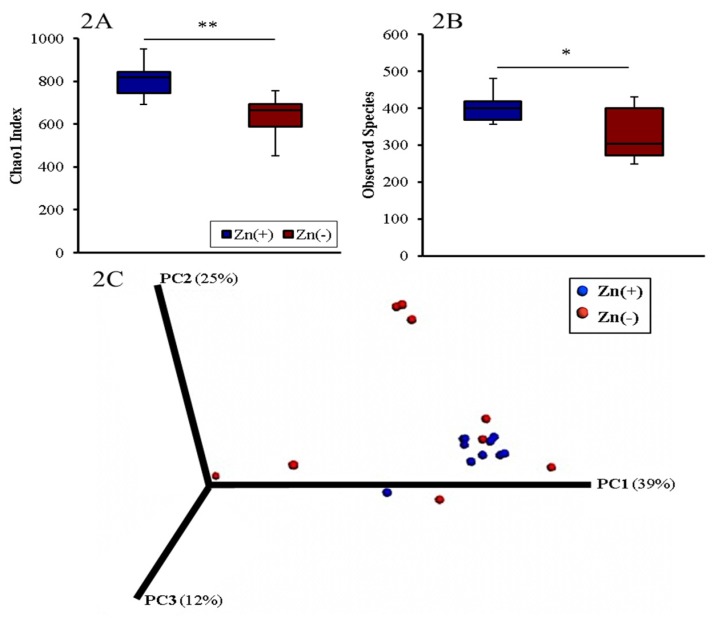
Microbial diversity of the cecal microbiome. (**A**) Measures of α-diversity using the Chao1 Index [39]; and (**B**) total number of observed species * *p* < 0.05, ** *p* < 0.01, ANOVA, *n* = 10 in Zn(+), *n* = 9 in Zn(−); (**C**) Measure of β-diversity using weighted UniFrac distances separated by the first three principal components (PC). Each dot represents one animal, and the colors represent the different treatment groups.

The diversity of the cecal microbiota in the Zn(+) and Zn(−) groups was assessed through measures of α–diversity, β–diversity, and overall species richness (Figure 2). The Chao1 index and observed species richness were used to assess α–diversity. For both measures, the Zn deficient group had significantly lower phylogenetic diversity, indicating a less diverse cecal microbial composition (Figure 2A,B). We utilized weighted UniFrac distances as a measure of β-diversity to assess the effect of chronic Zn deficiency on between-individual variation in bacterial community composition. Principal coordinate analysis demonstrated a significant expansion of β-diversity in the Zn deficient group (Figure 2C). Interestingly, the same features of lower α-diversity and richness together with higher β-diversity compared to the control as seen in Zn deficiency are also found in GI microbiota observed during a deficiency of the trace mineral selenium [36], as well as in various pathological states such as Crohn’s disease [37], inflammatory bowel disease [38], opportunistic infections [39], diabetes [40], obesity [41] and others [42].

### 3.3. Chronic Zn Deficiency Reshapes the Gut Microbiome

**Figure 3:**
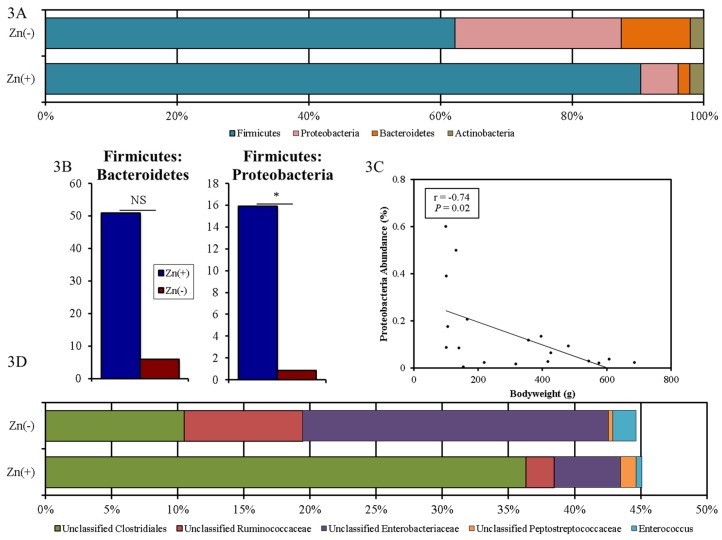
Phylum- and genera-level cecal microbiota shifts due to dietary Zn depletion. (**A**) Phylum-level changes between the Zn(+) and Zn(−) groups as measured at the end of the study (day 28). Only those phyla with abundance > 1% are shown; (**B**) Increased Firmicutes to Bacteroidetes and Proteobacteria ratios in the Zn(+) group (* *p* < 0.05, NS = not statistically significant); (**C**) Inverse correlation between Proteobacteria abundance and bodyweight; (**D**) Genus-level changes in the Zn(+) and Zn(−) group as measured at the end of the study (day 28). Only those genera significantly different between groups are shown.

We performed a taxon-based analysis of the cecal microbiota (Figure 3). 16S rRNA gene sequencing revealed that 98%–99% of all bacterial sequences in both the Zn(+) and Zn(−) groups belonged to four major divisions: Firmicutes, Proteobacteria, Bacteroidetes, and Actinobacteria. Bacterial community composition was altered in the Zn deficient group, where significantly greater abundance of Proteobacteria and significantly lower abundance of Firmicutes (Figure 3A) was observed. In the Zn(−) group, the abundance of Bacteroidetes was increased whereas Actinobacteria was diminished, albeit not significantly. As such, the ratio of Firmicutes: Proteobacteria, was significantly lower in the Zn deficient group (Figure 3B). Further, the abundance of Proteobacteria inversely correlated with bodyweight (Figure 3C). Because of the central importance of Zn in growth and development, bodyweight is often the first anthropometric measurement to respond to Zn depletion and to quantify risk of complications related to Zn deficiency [43]. It has been a consistently reliable indicator of low Zn intake and Zn status in multiple cohorts and experimental models, and has been used by numerous others to quantify suboptimal dietary Zn deficiency [44,45]. Likewise in this study, final bodyweight strongly correlated with final serum Zn (ρ = 0.84, *p* = 0.0012, Figure 1G). At the family-level, Peptostreptococcaceae and unclassified Clostridiales were significantly lower, whereas Enterococcaceae and Enterobacteriaceae were significantly enriched, in the Zn deficient group. At the genus-level, we observed that Zn deficient animals had significantly higher relative abundance of *Enterococcus*, unclassified *Enterobacteriaceae*, and unclassified *Ruminococcaceae*, and significantly lower relative abundance of unclassified *Clostridiales* and unclassified *Peptostreptococcaceae* compared with their Zn replete counterparts (Figure 3D).

**Figure 4:**
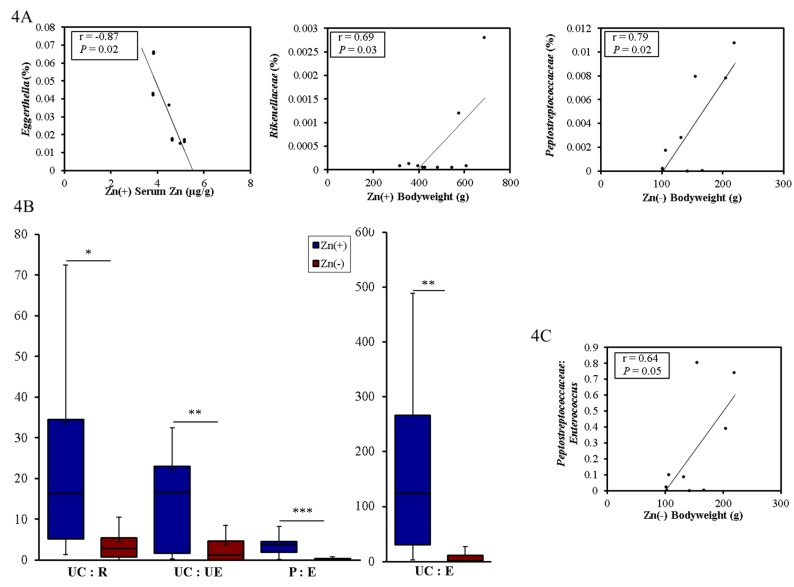
Genera–level correlations and ratios of cecal bacteria between the Zn(+) and Zn(−) groups. (**A**) Relative abundance of certain bacterial genera that significantly correlate with either treatment-specific serum Zn or bodyweight; (**B**) Ratios of bacterial genera that are significantly higher in the Zn(+) group (* *p* < 0.05, ** *p* < 0.01, *** *p* < 0.001, ANOVA); (**C**) Peptostreptococcaceae:Enterococcus ratio positively correlates with bodyweight in the Zn deficiency group.

We next investigated whether taxonomic shifts at the genus level were associated with host phenotype, as defined by bodyweight and serum Zn (as measured on day 28, Figure 4), two commonly utilized biomarkers of Zn deficiency. Among the Zn replete animals, a significant inverse correlation was obtained between average serum Zn levels and *Eggerthella* abundance. There was also a significant positive correlation between body weight and *Rikenellaceae* abundance in this group. In the Zn deficient group, a significant positive correlation was obtained between bodyweight and the abundance of *Peptostreptococcaceae.*

The ratio of certain bacterial groups may be predictive of shifts in the genetic capacity of the microbiome in certain physiological processes (e.g., the Firmicutes:Bacteroidetes ratio and caloric extraction from diet [46]. Studies have yet to characterize or relate taxonomic changes induced by dietary Zn deficiency to markers of the phenotype, yet such ratio analyses may further define a cecal microbiota signature of the deficiency. Our analysis revealed that several ratios of the significantly altered genera in the Zn(−) group were also significantly different during Zn deficiency. The ratios of the relative abundance of Unclassified *Clostridiales*:*Enterococcus* (UC:E), Unclassified *Clostridiales*:*Ruminococcaceae* (UC:R), Unclassified *Clostridiales*:Unclassified *Enterobacteriaceae* (UC:UE), and *Peptostreptococcaceae*:*Enteroccocus* (P:E) were significantly different between the Zn(+) and Zn(−) treatment groups (Figure 4B). Additionally, there was a significant, treatment–specific correlation between one of these ratios, *Peptostreptococcaceae*:*Enterococcus*, and bodyweight in the Zn deficient group (Figure 4C).

**Figure 5:**
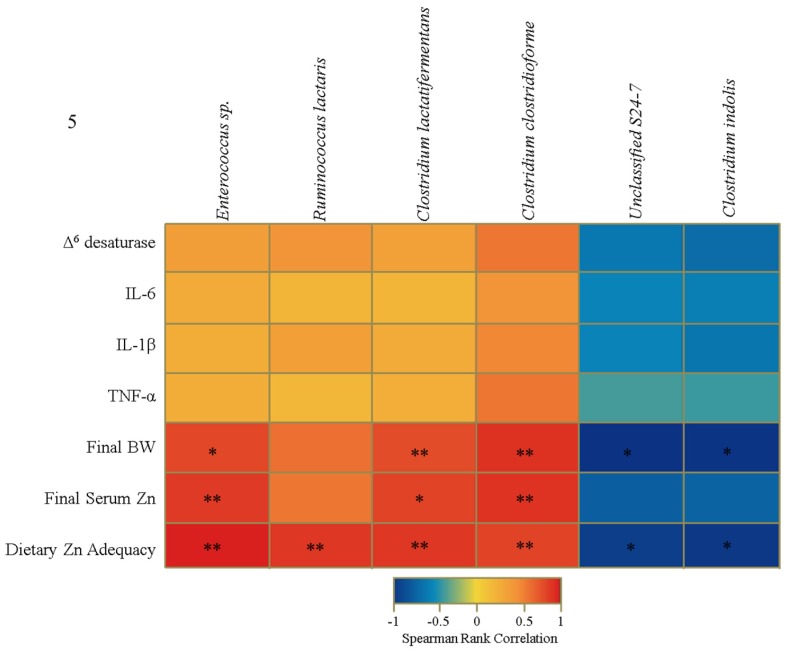
Heat map describing a set of Spearman correlations, independent of treatment group, between the relative abundance of different operational taxonomic units (OTUs) and select biological indicators of Zn status. The color indicator ranges from a perfect negative correlation (−1, blue) to a perfect positive correlation (1, red) (* *p* < 0.05, ** *p* < 0.01, ANOVA).

In light of these taxonomic alterations, we further analyzed community shifts to the species-level. We identified a strong positive correlation between *Ruminococcus lactaris*, *Enterococcus* sp., *Clostridium lactatifermentans*, and *Clostridium clostridioforme* and Zn adequacy, as well as between the latter three operational taxonomic units (OTUs) and final bodyweight and serum Zn measurements (Figure 5). The levels of two additional bacterial species, *Clostridium indolis* and an unclassified member of the Bacteroidales (*Unclassified S24*–*7*), were inversely correlated with final bodyweight and dietary Zn adequacy. Although not significant, it is interesting that the trend in correlation presented in Figure 5 (*i.e.*, positive OTU correlation with *Ruminococcus lactaris*, *Enterococcus* sp., *Clostridium lactatifermentans*, and *Clostridium clostridioforme* and negative OTU correlation with *Unclassified S24*–*7 and Clostridium indolis)* does extend to the mRNA gene expression data. 

### 3.4. Functional Alterations in the Genetic Capacity of Cecal Microbiota under Zn Deficiency Conditions

We next sought to understand whether the genetic capacity of the microbiota may influence host Zn status, since there were significant community shifts associated with physiological markers of Zn deficiency. The study of metagenomic alterations among various phenotypes (e.g., inflammatory bowel disease, obesity) and between healthy and diseased subjects has helped to elucidate how the functional shifts of the microbiota may affect the trajectory of the disease process [47]. However, the medical significance of alterations in the metabolic or functional capacity of the host microbiome under Zn deficiency conditions is unknown.

**Figure 6:**
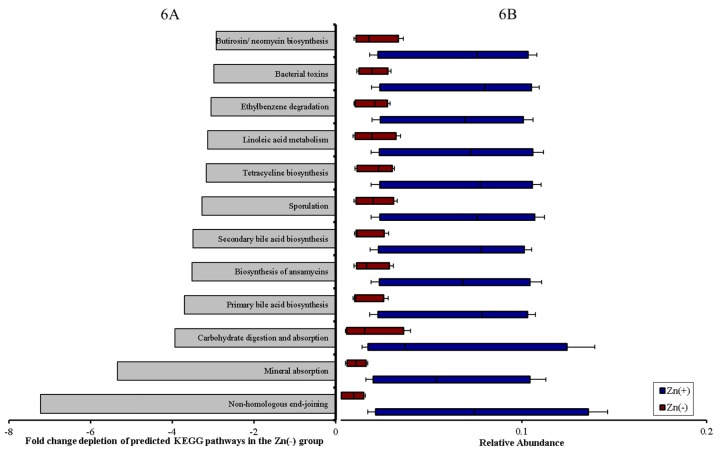
Functional capacity of the cecal microbiota is perturbed under conditions of Zn deficiency. (**A**) Fold change depletion of these pathways in the Zn(−) group (all *p* < 0.01, Student’s *t*–test); (**B**) Relative abundance of differentially–expressed KEGG microbial metabolic pathways in cecal microbiota. Treatment groups are indicated by the different colors (all *p* < 0.05, ANOVA).

Metagenome functional predictive analysis was carried out using PICRUSt software [48], OTU abundance was normalized by 16S rRNA gene copy number, identified using the Greengenes database, and Kyoto Encyclopedia of Genes and Genomes (KEGG) orthologs prediction was calculated [48]. Considering dietary Zn depletion was the singular variable in our experiments, 12 of the 265 (4.5%) KEGG metabolic pathways analyzed were differentially–expressed between the Zn deficient and adequate groups (Figure 6A,B). Non-homologous end–joining was most significantly depleted in Zn deficiency, an expected finding as Zn fingers are found in the catalytic subunit of DNA polymerase [49] and are essential for DNA binding and repair [50]. Further, we observed that even basic cecal microbiome metabolism was perturbed under Zn deficiency; pathways involving lipid metabolism, carbohydrate digestion and absorption, and, most pertinent to this study, mineral absorption were significantly depleted in the Zn(−) group. Other disruptions in microbial pathways involving the biosynthesis of bile acid and secondary metabolites, and xenobiotic detoxification reflect the fundamental requirement of dietary Zn in Zn finger motifs and in copper-zinc superoxide dismutase/glutathione enzymes, respectively. 

**Figure 7:**
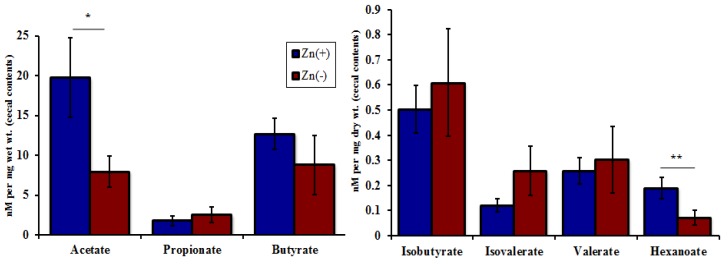
Concentration of short chain fatty acids (SCFAs) in the cecal contents in the Zn(+) and Zn(−) groups (* *p* < 0.05, ** *p* < 0.01, ANOVA).

Finally, we utilized a GC-MS (Gas chromatograph–mass spectrometer) to analyze SCFA concentration in the cecal contents of the Zn(−) and Zn(+) birds (Figure 7). SCFAs are produced by bacterial fermentation and serve as a primary metabolic substrate for colonocytes [51]. We observed a significant decrease in the concentration of acetate (C_2_) and hexanoate (C_6_) in Zn(−) cecal contents. Pertinent to our results, SCFAs may increase dietary Zn absorption via a decrease in luminal pH in the intestines [52], thereby increasing Zn solubility, and/or via stimulation of the proliferation of intestinal epithelial cells leading to an increase in the overall absorptive area of the intestines [53]. In this study, a decrease in SCFA concentration in the Zn(−) group may have followed from either the observed bacterial composition shifts and/or the decreased output of carbohydrate metabolism and fermentation via changes in microbial metabolic pathways. In the host, this may initiate a continuous cycle, which serves to limit Zn uptake even in an already Zn deficient state.

## 4. Discussion 

The gut microbial ecology is known to play a prominent role in host nutritional status, through mechanisms such as modulating saccharide cellular uptake [54], influencing energy balance [46], and *de novo* biosynthesis of particular vitamins and minerals [55]. The gut, which houses the majority of these microbes, is an important organ in the absorption of Zn from the diet [56,57,58]. Insufficient and/or poorly bioavailable dietary Zn intake are the primary etiological risk factors of Zn deficiency [59,60,61]. However, to our knowledge, the significance, if any, of compositional and/or functional changes in the gut microbiome during dietary Zn depletion has yet to be explored.

As is the case in humans and the vast majority of animals, *Gallus gallus* harbors a complex and dynamic gut microbiota [62], heavily influenced by host genetics, environment, and diet [63]. There is considerable similarity at the phylum level between the gut microbiota of broilers (*Gallus gallus*) and humans, with Bacteroidetes, Firmicutes, Proteobacteria, and Actinobacteria representing the four dominant bacterial phyla in both [54,64]. Due to its rapid maturation and well–characterized phenotype during mineral deficiency, *Gallus gallus* has been used extensively as a model of human nutrition, especially as it pertains to assessing physiological outcomes of low dietary Fe and Zn [65,66,67,68,69,70,71,72]. Therefore, a central aim of the present study was to use *Gallus gallus* as a model to characterize cecal bacterial community changes between Zn deficient and Zn replete groups. Our data demonstrate that in chronic Zn deficiency, species richness, as measured by the Chao1 index, and species diversity, as measured by the total observed OTUs, were both significantly decreased. Conversely, a significant increase in UniFrac distances was observed in the Zn deficient group, signifying the looser community relatedness of the cecal microbiomes of Zn deficient animals compared with their Zn replete counterparts. Since Zn is an essential mineral for many bacteria [73], we suggest that a Zn–depleted environment might lead to a less diverse community, preferentially composed of bacterial species that are viable under Zn–limiting conditions. These alterations in cecal microbiota diversity indices mirror those found in a range of GI [37,38] and non-GI disease states [39,40,41,42,74]. A similar dysbiotic profile has also been observed in the microbiota of micronutrient–deficient, malnourished children [75,76]. This pattern may exemplify the striking effect of suboptimal dietary Zn intake, as with other essential micronutrients, on bacterial diversity. Therefore, loss of global diversity of the cecal microbiota during Zn deficiency may be an important, yet non-specific, indicator of suboptimal Zn intake. 

Resident microbes of the gut microbiome compete with their host for various vitamins and transition elements [16,77,78,79], such as Fe and Zn. Particularly important, Zn ions are involved in numerous structural and catalytic proteins in most organisms, with Zn-binding proteins constituting 10% of the human proteome and nearly 5% of the bacterial proteome [80,81]. One form of host–microbe competition occurs through the encoding of bacterial transporters, such as the high–affinity Zn transporter, ZnuABC, in the bacterial genome, representing the essential nature of Zn for bacterial viability [77]. In our study, the compositional alterations in the Zn deficient group, most notably the significant expansion of the phylum Proteobacteria, as well as the genera *Enterobacteriaceae* and *Enterococcus*, may help to explain how dietary Zn and the microbiota interact, since the ZnuABC transporter has been found to be induced in many species within these bacterial groups under Zn-limiting conditions [82,83]. Lack of sufficient bioavailable dietary Zn in the lumen, therefore, may modulate the gut microbiota by enabling colonization and outgrowth of bacteria that can efficiently compete for Zn. Further, we postulate that microbe-microbe interactions through a decrease in the preponderance of members of the Firmicutes phylum such as the genus *Clostridium*, known SCFA producers, may explain the overgrowth of these bacteria in the Zn(−) group [84]. SCFAs have been shown to inhibit the growth of certain Proteobacteria such as members of the *Enterobacteriaceae* *in vivo* [84,85,86], and thus a decrease in SCFA concentration may further explain the cecal compositional shift observed during Zn deficiency. Additionally, alterations in the luminal environment of the intestines, such as a reduction in pH through increased SCFA production, can result in a notable increase in Zn bioavailability and uptake [57,87]. Therefore, our data suggest that changes in the gut microbiota composition of the Zn deficient group can further deplete Zn availability in an already Zn deficient state. Although we expected to observe a conservation of endogenous Zn through compensatory mechanisms in the Zn(−) group, upregulation of the expression of brush–border membrane proteins responsible for Zn uptake (*i.e.*, the ZnT and ZIP family transmembrane proteins) were not observed in the Zn(−) group [12]. Thus, our results suggest that the host-microbe balance may tilt in favor of the resident cecal microbiota (*i.e.,* the sequestration of Zn by the microbiota) during chronic Zn deficiency. 

As opposed to the competition–based mechanism underlying how altered Zn availability may structurally change the gut microbiota, a compensation-based mechanism may explain the metagenomic differences between the two groups. In the Zn deficient group, depletion of a key KEGG pathway, the mineral absorption pathway, was observed. The interplay between inadequate host Zn availability and commensal gut microbes may be implicated in the compensation for the relative lack of dietary Zn in the Zn(−) group; accordingly, this might lead to a depletion in bacterial pathways responsible for Zn uptake, and an enrichment in host mineral absorption pathways for the purpose of improving systemic Zn status. Additionally, lack of Zn available to the bacteria might also cause a decrease in bacterial Zn accumulation. 

Another aim of our study was to identify correlations between candidate microbes and commonly-used biological indicators of Zn deficiency (Figure 5C). *Clostridium indolis,* a microbe we found to be negatively correlated with bodyweight and Zn adequacy, has been isolated from clinical samples of both animal and human infections [88], and may have the potential to produce beneficial SCFAs such as acetate and butyrate [89]. *Enterococcus* sp. was positively correlated with final body weight, serum Zn, and Zn adequacy. Members of this genus, specifically *Enterococcus faecium,* have been shown previously to correlate with increased bodyweight [90] and elevated serum Fe levels [91]. The presence of *Clostridium lactatifermentans*, a SCFA producer, positively correlated with bodyweight, serum Zn, and Zn adequacy. It has been isolated previously from *Gallus gallus,* and associated with an improvement in growth and development (as defined by bodyweight) [92]. Aside from these studies, there are little data linking any of these microbes with a purported influence of host Zn status or overall physiology. Future research using GF animals may elucidate new roles for these specific microbes in the etiology and/or progression of Zn deficiency.

## 5. Conclusions

**Figure 8:**
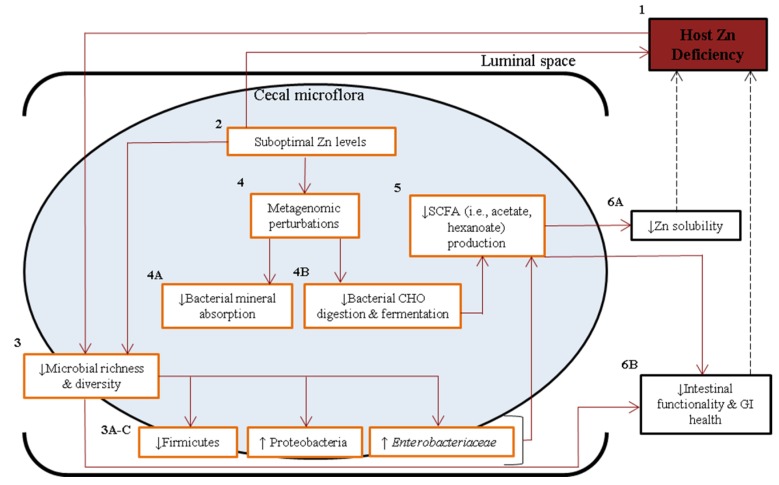
Schematic diagram depicting proposed mechanisms by which a Zn deficient gut microbiome may worsen a Zn deficient phenotype. Zn deficiency (1), caused by insufficient dietary Zn (2), induces a decrease in gut microbial diversity (3), and an outgrowth of bacteria particularly suited to low Zn conditions, leading to dysbiosis [3A–C]. Lack of dietary Zn also leads to alterations in the functional capacity of the microflora (4), causing multiple effects including decreased expression of pathways related to mineral (*i.e.*, Zn) absorption (4A) and carbohydrate digestion and fermentation (4B). A decrease in the latter pathway may also cause a depression in the production of SCFAs (5), compounds responsible for improving the bioavailability of Zn. Altogether, these microbial effects may decrease Zn absorbability (6A, [87]) and disturb GI health (6B, [93,94]), thereby perpetuating a Zn deficient state. Red arrows and orange–lined boxes denote observations of this study, and dashed arrows and black–lined boxes describe published findings.

We have revealed a dramatic compositional and functional remodeling that occurs in the *Gallus gallus* gut microbiota under chronic Zn deficient conditions. Compositional alterations in bacterial abundance, in part due to host–microbe and microbe–microbe interactions, lead to changes in the functional capacity of the microbiota, such as SCFA output, which can influence the absorption and availability of dietary Zn by the host. Our data suggest that as a consequence of this remodeling, a Zn (–) microbiota has the potential to perpetuate, and perhaps even aggravate, the Zn deficient condition through the further sequestration of Zn from the host (Figure 8). Such a microbiota are not functionally compatible with the physiological needs of the Zn deficient host. In addition, others have observed decreased luminal Zn solubility in the intestines [87], increased GI inflammation and intestinal permeability, and an overall decline in GI health [93,94] under Zn deficiency. Our findings add to this knowledge by suggesting possible mechanisms by which the gut microbiota may contribute to host Zn deficiency. Further research should determine whether the gut microbiome could represent a modifiable risk factor for chronic Zn deficiency.

## Supplementary Materials

The following are available online at http://www.mdpi.com/2072-6643/7/12/5497/s1, Table S1: Composition of the experimental diets, Table S2: Measured genes (*Gallus gallus*) and tissue-specific 18S rRNA from mRNA.
