# Supplementary material for: Chronic Zinc Deficiency Alters Chick Gut Microbiota Composition and Function
Source: Nutrients. 2015 Nov 27;7(12):9768–84. doi: 10.3390/nu7125497 (PMC4690049; doi:10.3390/nu7125497)
Supplement: Supplementary file 1 [file nutrients-07-05497-s001.docx]

Supplementary Materials: Chronic Zinc Deficiency Alters Chick Gut Microbiota Composition and Function

Spenser Reed ^1,2,3^, Hadar Neuman ^4^, Sharon Moscovich ^4^, Raymond P. Glahn ^1^, Omry Koren ^4^ and Elad Tako ^1,^*

1. Animals, Diets, and Experimental Design

Fertile Cornish–cross broiler eggs were obtained from a commercial hatchery (Moyer’s Chicks, Quakertown, PA, USA). The eggs were incubated under optimal conditions at the Cornell University Animal Science Poultry Farm incubator. Upon hatching (hatchability rate was 94%), chicks were randomly allocated into two treatment groups on the basis of body weight and gender (aimed to ensure equal distribution between groups, *n* = 12): 1. Zn(+): 42 µg/g zinc; 2. Zn(−): 2.5 µg/g zinc. The experimental diets, including Zn levels, were designed based on the NRC recommendations and requirements for Poultry [1]. Experimental diets differed only in levels of supplemental Zn (as Zn carbonate). Chicks were housed in a total–confinement building (one chick per 0.5 m^2^ metal cage). Birds were under indoor controlled temperatures and were provided 16 h of light. Each cage was equipped with an automatic nipple drinker and manual self–feeder. All birds were given *ad libitum* access to water and food. At study conclusion (day 28) birds were euthanized by CO_2_ exposure. The digestive tracts (colon and small intestine) and liver were quickly removed from the carcass and separated into various sections for tissue analysis (~1–2 cm; ~2–3 g was taken from small intestine and liver, respectively). Before the study conclusion, 3 birds in the Zn(−) group died which brought the sample size of this group to *n* = 9. The samples were immediately frozen in liquid nitrogen, and then stored in a −80 °C freezer until analysis. All animals were hatched at the same facility. All animal protocols were approved by the Cornell University Institutional Animal Care and Use committee.

2. Determination of Zn Status

Blood samples were collected weekly from the wing vein (*n* = 12, ~100 µL) using micro-hematocrit heparinized capillary tubes (*Fisher Scientific*, Pittsburgh, PA, USA). Samples were collected in the morning following an 8 h overnight fast. Serum Zn. Serum Zn concentrations were determined by an inductively-coupled argon-plasma/atomic emission spectrophotometer (ICAP 61E Thermal Jarrell Ash Trace Analyzer, Jarrell Ash Co., Franklin, MA, USA) following wet ashing. Erythrocyte fatty acid analysis (LA:DGLA Ratio). The LA:DGLA ratio is a novel biomarker reflecting dietary Zn intake [2]. Red blood cell fatty acids were determined as described [2]. Gene Expression Markers. PCR was carried out as previously described [2–7]. Tissue-specific 18S rRNA was used to normalize the results. All PCR products were separated by electrophoresis on a 2% agarose gel, stained with ethidium bromide, and quantified using Quantity One 1-D analysis software (Bio-Rad, Hercules, CA, USA). Values are reported in arbitrary units (AU).

3. Isolation of Total RNA

Total RNA was extracted from 30 mg of duodenal (proximal duodenum, *n* = 9) and liver tissues (*n* = 9) using Qiagen RNeasy Mini Kit (Qiagen Inc.*,* Valencia, CA, USA) according to the manufacturer’s protocol. All steps were carried out under RNase free conditions. RNA was quantified by absorbance at 260–280 nm. Integrity of the 28S and 18S rRNA was verified by 1.5% agarose gel electrophoresis followed by ethidium bromide staining.

4. Cecal SCFA Analysis

SCFA concentration was determined as previously described [8]. Briefly, aliquots of dried cecal samples (about 100 mg) were homogenized after the addition of 600 μL of cold extraction solution containing internal standard (IS). Samples were then centrifuged at 15,000 *g* for 10 min at 4 °C. Next, 300 μL of cold diethyl ether was added to 300 μL of supernatant. After a 5 min incubation, samples were centrifuged at 15,000 *g* for 1 min at 4 °C. The upper layer was then transferred to an autosampler vial with an insert, and immediately capped. Calibration curves were prepared adding the IS to scalar amounts of the acids in diluted samples or water (for external standardization). Quantification was carried out through the use of extracted ion chromatograms by selecting fragment ions of the studied analytes. The SCFA concentration in cecal samples was expressed as nM mg^−1^.

5. 16S rRNA PCR (Polymerase Chain Reaction) Amplification and Sequencing

Microbial genomic DNA was extracted from cecal samples using the PowerSoil DNA isolation kit, as described by the manufacturer (MoBio Laboratories Ltd, Carlsbad, CA, USA). Bacterial 16S rRNA gene sequences were PCR-amplified from each sample using the 515F-806R primers for the V4 hypervariable region of the 16S rRNA gene, including 12-base barcodes, as previously described [9]. PCR reactions consisted of 12.5 µL KAPA HiFi HotStart ReadyMix (kit KK2601, Kapa Biosystems, Woburn, MA, USA), 10 µM of each primer, and 10–100 ng DNA template. Reaction conditions consisted of an initial denaturing step for 3 min at 95 °C followed by 31 cycles of 20 s at 98 °C, 15 s at 60 °C and 20 s at 72 °C. Triplicate PCR reactions were performed for each sample, which were combined and then purified with Ampure magnetic purification beads (Agencourt, Danvers, MA, USA). Purified PCR products were quantified using a Quant–iT PicoGreen dsDNA assay (Invitrogen, Carlsbad, CA, USA). Equimolar ratios of total samples were pooled and sequenced at the Faculty of Medicine of the Bar Ilan University (Safed, Israel) using a MiSeq Sequencer (Illumina, Madison, WI, USA).

6. 16S rRNA Gene Sequence Analysis

For quality filtering of raw data, sequences with Phred < 20, or shorter than 75% of expected length were discarded, as well as sequences containing primer mismatches, uncorrectable barcodes, ambiguous bases, or homopolymer runs in excess of 6 bases. The sequences that passed the quality filters were analyzed using the QIIME software package [10]. Sequences were classified taxonomically using the Greengenes (GG) reference database at a confidence threshold of 80% [11]. The GG taxonomies were used to generate summaries of the taxonomic distributions of OTUs across different levels (phylum, order, family, and genus). To standardize sequence counts across samples with uneven sampling, we randomly selected 22,450 sequences per sample (rarefaction) and used this as a basis to compare abundances of OTUs across samples. For phylogenetic tree-based analyses, each OTU was represented by a single sequence that was aligned using PyNAST [12]. A phylogenetic tree was built with Fast-Tree [13] and used for estimates of α-diversity (within sample diversity, using Faith’s phylogenetic diversity [14]) and β-diversity (between sample diversity, using unweighted and weighted UniFrac [15]). For PD measurements, means and standard errors for given categories were calculated from 100 iterations using a rarefaction of 16,837 sequences per sample. Metagenome functional predictive analysis was carried out using PICRUSt software [16]. Briefly, OTU abundance was normalized by 16S rRNA gene copy number, identified and compared to a phylogenetic reference tree using the Greengenes database, and was assigned functional traits and abundance based on known genomes and prediction using the Kyoto Encyclopedia of Genes and Genomes (KEGG) [16]. Data representing significant fold-change differences in functional pathways between experimental groups was plotted.

**Table S1.** Composition of the experimental diets.

| **Ingredient** | **Zn(+) Diet** | **Zn(−) Diet** |
| --- | --- | --- |
| g/kg (by formulation) |  |  |
| Egg whites | 200 | 200 |
| dl-Methionine | 3 | 3 |
| Cornstarch | 318.2 | 318.2 |
| Dyetrose | 105 | 105 |
| Dextrose | 200 | 190.8 |
| Cellulose | 50 | 50 |
| Corn oil | 50 | 50 |
| Salt mix (no Zn) | 60 | 60 |
| Vitamin mix | 10 | 10 |
| Biotin (1 mg/g) | 1.8 | 1.8 |
| Choline bitartrate | 2 | 2 |
| Zinc carbonate (5 mg/g) | 9.2 | − |
| Total (g) | 1000 | 1000 |
| Concentrations of Selected Components (means ± SEM, *n* = 5) | | |
| Zinc concentration (ppm) ** | 42.29 a ± 0.25 | 2.55 b ± 0.02 |
| Iron concentration (ppm) Phytic acid | 98.75 ± 2.04 | 102.19 ± 5.21 |
|  | <dl | <dl |

* Modified NRC [1] purified chicken diets were provided by Dyets Inc. Bethlehem, PA (Zn(+), Zn adequate control diet: 135251, Zn(−), Zn deficient diet: 135252); ** Determination of zinc concentration is described in the materials and methods section; a, b Within a column, means without a common letter are significantly different (*p* < 0.05).

**Table S2.** Measured genes (*Gallus gallus*) and tissue-specific 18S rRNA from mRNA.

| **Analyte** | **Organ** | **Forward Primer (5′à3′) (Nucleotide Position)** | **Reverse Primer (5′à3′) Length** | **(Nucleotide Position)  (Base Pairs)** | **GI Identifier** |
| --- | --- | --- | --- | --- | --- |
| ZnT1 | Intestine | CCTCCAGACAACCTTTGGTG (64–83) | TACTGATCTGCAAACCTTGCCA (133–112) | 69 | 54109718 |
| ZnT5 | Intestine | TCGTGGAGGCTGTCATTCAC (1657–1676) | TGCAGATCTTTCTCCTGTTCGT (2016–1995) | 359 | 56555150 |
| ZnT7 | Intestine | GGCGTCTGGAGTAACAGCTT (166–185) | GTGAATGCCCATGACCTCCA (502–483) | 336 | 56555152 |
| ZIP6 | Intestine | TTGTGGAATCATCCCAGGGC (549–568) | GCTCATTCGCATCTCTCCGA (929–909) | 380 | 66735072 |
| ZIP9 | Intestine | TTATTCCCCTGGCCGTGAAC (68–87) | CCAATGCGAAGACCAGCAAG (643–624) | 575 | 237874618 |
| TNF-α | Liver | CATTTGGAAGCAGCGTTCGG (48–67) | GACAGGGTAGGGGTGAGGAT (249–230) | 202 | 53854909 |
| IL-1β | Liver | CCTCCAGCCAGAAAGTGAGG (431–450) | TTGTAGCCCTTGATGCCCAG (539–520) | 109 | 88702685 |
| IL-6 | Liver | AACAACCTCAACCTGCCCAA (338–357) | AGGTCTGAAAGGCGAACAGG (449–430) | 112 | 302315692 |
| NF-κB | Liver | GGATGGTCTGTTCCTGAAGA (1682–1702) | ACCTCTGCCTGCTTTGTGAT (1981– 1961) | 300 | 2130627 |
| AP | Intestine | GAATGAGGGCTTTGCCTCCT (1245–1264) | GAAGTTGCTGTTGGTGGCTG (1854–1835) | 610 | 45382360 |
| SI | Intestine | CAGATCTCAGCCCGTCTTCC (237–256) | CCAGAATGCCACCGGTAACT (519–500) | 282 | 2246388 |
| Na+ K+ ATPase | Intestine | CTGAGGGCAACGAAACAGTG (104–123) | ATCCCTCGGGTTGACCTCC (177–159) | 74 | 14330321 |
| SGLT-1 | Intestine | GTGGAATGCCTTGGAGGGTA (3–22) | GCTTCCTCAGATACTCCGGC (123–104) | 121 | 8346783 |
| MT4 | Intestine | ACCCGAACTGAACCATGGAC (36–55) | TTTTCGTGGTCCCTGTCACC (312–293) | 277 | 46048710 |
| Δ6 desaturase | Liver | ACATGAACAGAGGAAGCGGG (780–799) | TCTGGATCTCCTCCCAGGTG (1754–1735) | 975 | 261865208 |
| DMT-1 | Intestine | TTCCTCCTCAACAACGTCGG (1755–1774) | TCCCAATGCCATCCCAGTTC (1908–1889) | 154 | 206597489 |
| 18S rRNA | Intestine, Liver | CGATGCTCTTAACTGAGT (1251–1269) | CAGCTTTGCAACCATACTC (1550– 1531) | 300 | 7262899 |

Reference

1. Subcommittee on Poultry Nutrition; Committee on Animal Nutrition; Board on Agriculture; National Research Council. *Nutrient Requirements of Poultry*; National Academies Press: Washington, DC, USA, 1994.
2. Reed, S.; Qin, X.; Ran-Ressler, R.; Brenna, J.T.; Glahn, R.P.; Tako, E. Dietary zinc deficiency affects blood linoleic acid: dihomo-ihlinolenic acid (LA:DGLA) ratio; a sensitive physiological marker of zinc status *in vivo* (*Gallus gallus*). *Nutrients* **2014**, *6*, 1164–1180.
3. Mahler, G.J.; Esch, M.B.;Tako, E.; Southard, T.L.; Archer, S.D.; Glahn, R.P.; Shuler, M.L. Oral exposure to polystyrene nanoparticles affects iron absorption. *Nat. Nanotechnol.* **2012**, *7*, 264–271.
4. Tako, E.; Rutzke, M.A.; Glahn, R. Using the domestic chicken (*Gallus gallus*) as an *in vivo* model for iron bioavailability. *Poult. Sci.* **2010**, *89*, 514–521.
5. Tako, E.; Glahn, R.P. White beans provide more bioavailable iron than red beans: Studies in poultry (*Gallus gallus*) and an *in vitro* digestion/Caco-2 model. *Int. J. Vitam. Nutr. Res.* **2010**, *80*, 416–429.
6. Tako, E.; Blair, M.; Glahn, R.P. Biofortified red mottled beans (*Phaseolus vulgaris* L.) in a maize and bean diet provide more bioavailable iron than standard red mottled beans: Studies in poultry (*Gallus gallus*) and an *in vitro* digestion/Caco-2 model. *Nutr. J*. **2011**, *10*, 113.
7. Tako, E.; Hoekenga, O.; Kochian, L.V.; Glahn, R.P. High bioavailability iron maize (*Zea mays* L.) developed through molecular breeding provides more absorbable iron *in vitro* (Caco-2 model) and *in vivo* (*Gallus gallus*). *Nutr. J*. **2013**, *12*, 3.
8. Schnorr, S.L.; Candela, M.; Rampelli, S.; Centanni, M.; Consolandi, C.; Basaglia, G.; Turroni, S.; Biagi, E.; Peano, C.; Severgnini, M.; *et al*. Gut microbiome of the Hazda hunter-gathers. *Nat. Commun.* **2014**, doi:10.1038/ncomms4654.
9. Caporaso, J.G.; Lauber, C.L.; Walters, W.A.; Berg-Lyons, D.; Huntley, J.; Fierer, N.; Owens, S.M.; Betley, J.; Fraser, L.; Bauer, M.; *et al*. Ultra-high-throughput microbial community analysis on the Illumina HiSeq and MiSeq platforms. *ISME J.* **2012**, *6*, 1621–1624.
10. Caporaso, J.G.; Kuczynski, J.; Stombaugh, J.; Bittinger, K.; Bushman, F.D.; Costello, E.K.; Fierer, N.; Peña, A.G.; Goodrich, J.K.; Gordon, J.I.; *et al*. QIIME allows analysis of high-throughput community sequencing data. *Nat. Methods* **2010**, *7*, 335–336.
11. McDonald, D.; Price, M.N.; Goodrich, J.; Nawrocki, E.P.; DeSantis, T.Z.; Probst, A.; Andersen, G.L.; Knight, R.; Hugenholtz, P. An improved Greengenes taxonomy with explicit ranks for ecological and evolutionary analyses of bacteria and archaea. *ISME J.* **2012**, *6*, 610–618.
12. Caporaso, J.G.; Bittinger, K.; Bushman, F.D.; DeSantis, T.Z.; Andersen, G..; Knight, R. PyNAST: A flexible tool for aligning sequences to a template alignment. *Bioinformatics* **2010**, *26*, 266–267.
13. Price, M.N.; Dehal, P.S.; Arkin, A.P. FastTree: Computing large minimum evolution trees with profiles instead of a distance matrix. *Mol. Biol. Evol.* **2009**, *26*, 1641–1650.
14. Faith, D.P. Conservation evaluations and phylogenetic diversity. *Biol. Conserv.* **1992**, *61*, 1–10.
15. Lozupone, C.; Knight, R. UniFrac: A new phylogenetic method for comparing microbial communities. *Appl. Environ. Microbiol.* **2005**, *71*, 8228–8235.
16. Langille, M.G.; Zaneveld, J.; Caporaso, J.G.; McDonald, D.; Knights, D.; Reyes, J.A.; Clemente, J.C.; Burkepile, D.E.; Vega Thurber, R.L.; Knight, R.; *et al*. Predictive functional profiling of microbial communities using 16S rRNA marker gene sequences. *Nat. Biotechnol.* **2013**, *31*, 814–821.
